# Combination of indirect revascularization and endothelial progenitor cell transplantation improved cerebral perfusion and ameliorated tauopathy in a rat model of bilateral ICA ligation

**DOI:** 10.1186/s13287-022-03196-1

**Published:** 2022-11-12

**Authors:** Kuo-Chuan Wang, Ling-Yu Yang, Jing-Er Lee, Vicent Wu, Te-Fu Chen, Sung-Tsang Hsieh, Meng-Fai Kuo

**Affiliations:** 1 Division of Neurosurgery, Department of Surgery, National Taiwan University Hospital, National Taiwan University College of Medicine, 7 Chun-Shan South Road, Taipei, 100 Taiwan; 2 Department of Neurology, Taipei Medical University-Wan Fang Hospital, Taipei, Taiwan; 3 Division of Nephrology, Department of Internal Medicine, National Taiwan University Hospital, Taipei, Taiwan; 4 Non-Invasive Cancer Therapy Research Institute, Taipei, Taiwan; 5 Department of Neurology, National Taiwan University Hospital, Taipei, Taiwan; 6 Department of Anatomy and Cell Biology, National Taiwan University College of Medicine, Taipei, Taiwan

**Keywords:** Endothelial progenitor cells, Microcirculation, Neuronal damage, Indirect revascularization, Angiogenesis, Cerebral ischemia

## Abstract

**Objective:**

Endothelial progenitor cells (EPCs) contribute to the recovery of neurological function after ischemic stroke. Indirect revascularization has exhibited promising effects in the treatment of cerebral ischemia related to moyamoya disease and intracranial atherosclerotic disease. The role of EPCs in augmenting the revascularization effect is not clear. In this study, we investigated the therapeutic effects of indirect revascularization combined with EPC transplantation in rats with chronic cerebral ischemia.

**Methods:**

Chronic cerebral ischemia was induced by bilateral internal carotid artery ligation (BICAL) in rats, and indirect revascularization by encephalo-myo-synangiosis (EMS) was performed 1 week later. During the EMS procedure, intramuscular injection of EPCs and the addition of stromal cell-derived factor 1 (SDF-1), and AMD3100, an SDF-1 inhibitor, were undertaken, respectively, to investigate their effects on indirect revascularization. Two weeks later, the cortical microcirculation, neuronal damage, and functional outcome were evaluated according to the microvasculature density and partial pressure of brain tissue oxygen (PbtO_2_), regional blood flow, expression of phosphorylated Tau (pTau), TUNEL staining and the rotarod performance test, respectively.

**Results:**

The cortical microcirculation, according to PbtO_2_ and regional blood flow, was impaired 3 weeks after BICAL. These impairments were improved by the EMS procedure. The regional blood flow was further increased by the addition of SDF-1 and decreased by the addition of AMD3100. Intramuscular injection of EPCs further increased the regional blood flow as compared with the EMS group. The rotarod test results showed that the functional outcome was best in the EMS combined with EPC injection group. Western blot analysis showed that the EMS combined with EPC treatment group had significantly decreased expressions of phosphorylated Tau and phosphorylated glycogen synthase kinase 3 beta (Y216 of GSK-3β). pTau and TUNEL-positive cells were markedly increased at 3 weeks after BICAL induction. Furthermore, the groups treated with EMS combined with SDF-1 or EPCs exhibited marked decreases in the pTau expression and TUNEL-positive cells, whereas AMD3100 treatment increased TUNEL-positive cells.

**Conclusion:**

The results of this study suggested that indirect revascularization ameliorated the cerebral ischemic changes. EPCs played a key role in augmenting the effect of indirect revascularization in the treatment of chronic cerebral ischemia.

**Supplementary Information:**

The online version contains supplementary material available at 10.1186/s13287-022-03196-1.

## Introduction

Cerebral ischemia leads to a limited oxygen supply to the brain. As the cerebrovascular disease progresses, the intracranial blood vessels will gradually become narrow or even occluded, resulting in a decrease in cerebral perfusion. Chronic cerebral hypoperfusion may lead to hyperphosphorylation of Tau protein and increased Aβ deposition or neuronal death, which are likely to cause motor and cognitive dysfunction [1, 2].

Endothelial progenitor cells (EPCs), which are thought to originate from bone marrow and can be isolated from adult peripheral or umbilical cord blood, have been implicated in vascular regeneration [3]. Evidence has shown that increased levels of EPCs are associated with reduced risks of cardiovascular events and endothelial dysfunction [3–5]. EPCs have also been implicated in angiogenesis after tissue ischemia [6, 7] and are capable of proliferating and differentiating into endothelial cells for vascular regeneration [8]. Indirect revascularization, such as encephalo-duro-arterio-synangiosis (EDAS) and encephalo-myo-synangiosis (EMS), has been used to treat pediatric and adult moyamoya disease (MMD) [9, 10]. The application of indirect revascularization was extended to the treatment of selected patients with intracranial atherosclerotic disease [11, 12]. The effect of EPCs on the angiogenesis induced by indirect revascularization is still not clear. Moreover, evidence is accumulating to show that stromal cell-derived factor-1 (SDF-1) plays an important role in the recruitment, proliferation and maturation of EPCs in the ischemic regions [13, 14]. The level of SDF-1 has been also demonstrated to be strongly associated with the number of EPCs and the acute lesion volume in ischemic stroke patients [15].

Bilateral internal carotid artery ligation (BICAL) has been widely used to impair brain vasculature in experimental animals [16]. In the present study, we established a BICAL rat model and performed EMS, an indirect revascularization procedure, to imitate the treatment of synangiosis in chronic ischemic human brains. The role of EPCs in modulating the effects of EMS in the chronic ischemic brain was investigated. In addition to cortical microcirculation changes, the functional outcome and neuronal damage, and associated factors were studied in the chronic ischemic rats with and without various treatments with EPCs after indirect revascularization was performed.

## Materials and methods

This study was approved by the Institutional Animal Care and Use Committee of National Taiwan University and was conducted in accordance with ethical regulations (Approval no. IACUC-20180430). All surgical procedures were performed according to the Guide for the Care and Use of Laboratory Animals (issued by the National Institutes of Health) and approved by the Committee.

### Experimental design

**Fig. 1:**
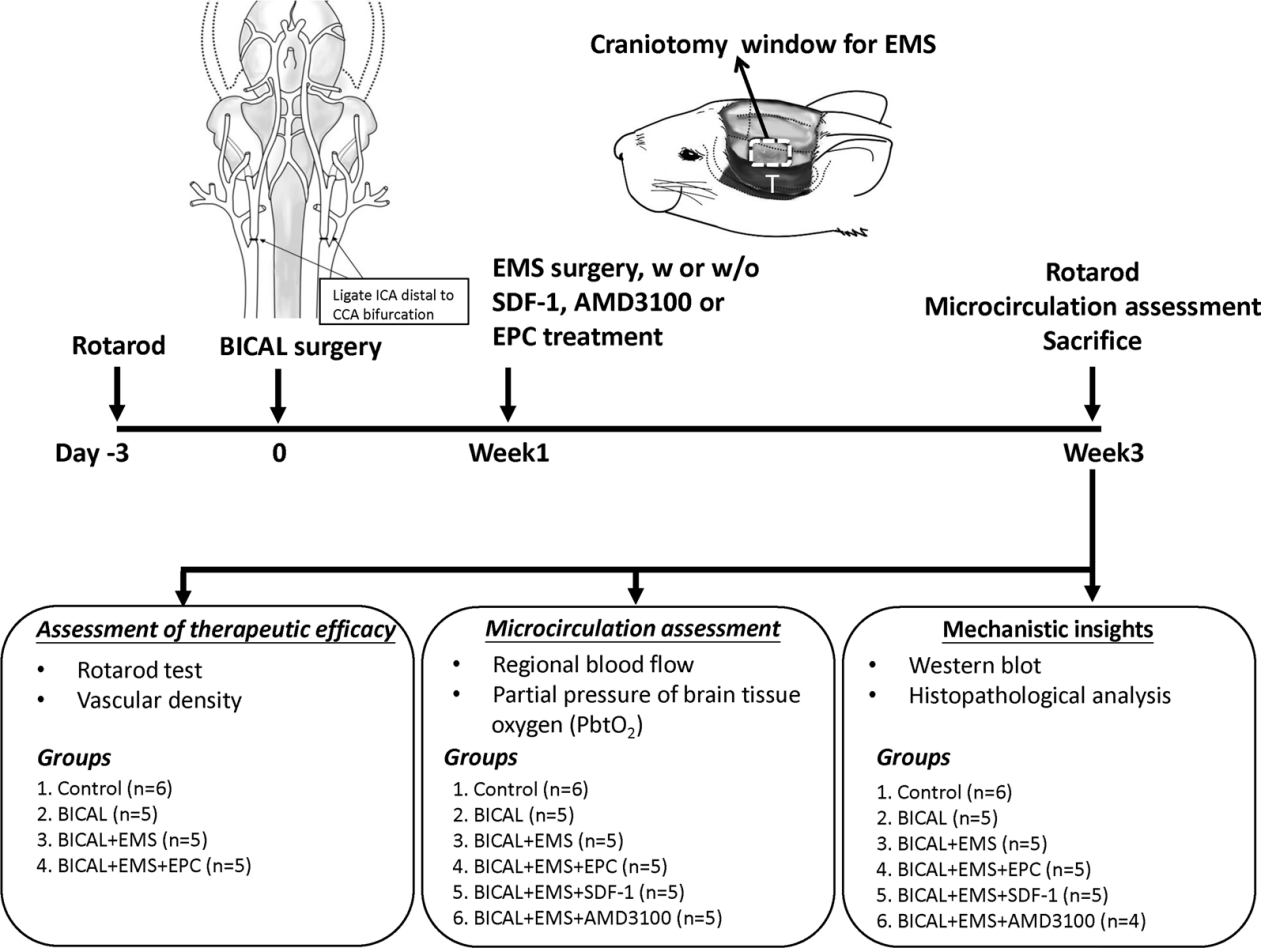
Schematic representation of the experimental procedure. BICAL surgery was performed by permanent ligation of the ICA distal to the bifurcation of the CCA. One week following BICAL surgery, the craniotomy window for EMS (white dotted box) was created after downward reflection of the temporalis muscle (marked as T). Treatment with EPCs, SDF-1 or AMD3100 was administered during the EMS procedure. The rotarod test was performed 3 days before and 3 weeks after BICAL. Vascular density and microcirculation were evaluated 3 weeks after BICAL. Animals were killed 3 weeks following BICAL surgery, and brain tissues were harvested for Western blot analysis and histopathological analysis

Figure 1 shows a schematic plot of the experimental design. Male Wistar rats (each weighing 200–250 g) were randomly assigned to the various treatment groups. The researchers were blinded to treatment allocation through the entire duration of the study. The rats were anesthetized with an intraperitoneal injection of Zoletil 50 mg/kg and Xylazine 8 mg/kg, and intraperitoneal premedication with atropine sulfate 0.05 mg/kg in 1 ml 0.9% NaCl to reduce hypersalivation. Deep sedation was verified as the absence of hind- and fore-limb pain reflexes, as well as the absence of corneal reflexes. Normal non-labored breathing was maintained throughout the surgery. Body temperature was monitored with a rectal probe and maintained at 37 °C by placing the animals on a thermal blanket. Blood pressure was measured via an arterial line and maintained at 100–120 mmHg during the procedures of BICAL, EMS, and microcirculation measurement by controlling the depth of anesthesia and volume replacement. Rats that had died or had severe weight loss (above 20%) within 7 days after BICAL surgery were also excluded.

To test the therapeutic effects of EPCs, 23 rats were used in the first set. By the exclusion criteria, died animals were excluded (mortality rate was 8.69%, 2/23). The first set of rats underwent BICAL followed by EMS surgery or EMS + EPC at 7 days after BICAL. Three weeks after BICAL surgery, these rats were used for behavioral analyses and microvasculature density counting (*n* = 5–6).

To evaluate microcirculation in the brain cortex, the second set of rats underwent BICAL followed by EMS surgery, EMS + SDF − 1, EMS + AMD3100 or EMS + EPC at 7 days after BICAL (mortality rate was 8.82%, 3/34). Three weeks after BICAL surgery, these rats were used for microcirculation assessment (*n* = 5–6).

To study mechanistic aspects of EPCs function, the third set of rats were divided into groups as the second set (mortality rate was 11.76%, 4/34). Three weeks after BICAL surgery, these rats were used for Western blot analysis, and histopathological studies of brain injury (*n* = 4–6). Number of rats in each group separately used for assessment of each set is displayed in Fig. 1.

### Induction of chronic cerebral ischemia: bilateral internal carotid artery ligation (BICAL)

A longitudinal incision was made in the midline of the neck. The common carotid artery (CCA) bifurcation was carefully dissected, and then, the internal carotid artery (ICA) distal to the bifurcation was ligated (Fig. 1). To avoid the acute deficit in cerebral perfusion of the rats, we use a 30 min delayed ligation between ICAs to decrease mortality, as described in previously study [17].

### An indirect revascularization model in rats: encephalo-myo-synangiosis (EMS)

One week after BICAL, the temporalis muscle was detached from the cranium to the zygoma on the left side. A 5 × 5 mm craniectomy underneath the temporalis muscle was performed, and the exposed dura was excised. 0.2 mL of TISSEEL® (0.1 mL of fibrinogen and 0.1 mL of thrombin) (Baxter Healthcare Corporation) was applied onto the exposed brain surface with dura mater opening; then, the temporalis muscle was placed back in contact with the TISSEEL and the brain surface. Light pressure was maintained on the muscle for 2 min, and excess TISSEEL was squeezed out and removed. The wound was closed after hemostasis was achieved.

### Addition of SDF-1 or AMD3100 and intramuscular injection of EPCs during EMS procedure

#### Preparation of SDF-1 and AMD3100 gel

In the experimental groups, SDF-1 and AMD3100, an SDF1 antagonist, were dissolved, respectively, in thrombin (200 ng/cc thrombin), one component of TISSEEL, then mixed with the other component of TISSEEL, fibrinogen. The SDF-1- or AMD3100-containing TISSEEL was used as a sealant during the EMS procedure.

#### Preparation of EPCs and intramuscular injection of EPCs during EMS procedure

The following study was approved by the Institutional Review Board of the National Taiwan University Hospital, Taipei, Taiwan. With the consent of the parents, fresh human cord blood was obtained after birth. Preparation of EPCs was performed as described previously [18]. Briefly, whole blood was isolated by density centrifugation over Ficoll-Paque PLUS (GE Healthcare). The mononuclear cell (MNC) layer was then collected and the cells counted. Eighty-million cells were seeded over one fibronectin-coated (10 μg/mL) 100-mm Petri dish with endothelial cell basal medium-2 (EBM-2) (Lonza) supplemented with endothelial cell growth medium-2 (EGM-2) MV SingleQuots (Lonza) containing FBS (20%). The medium was changed every 3 days until completion of differentiation had been established by morphology; the cells were characterized as endothelial cell-like by immunocytochemistry of CD31 (Abcam, #ab28364, 1:50), VE-cadherin (Cell Signaling, #2158, 1:100), and VEGF receptor 2 (Cell Signaling, #2479, 1:200), but negative for CD133 (Abcam, #ab19898, 1:200), then employed in our study. EPCs were cultured for 3–4 passages, detached using trypsin–EDTA and washed twice in PBS. 2 × 10^6^ EPCs were counted and re-suspended in 25 μL phosphate-buffered saline (PBS). EPCs were injected into the temporalis muscle using separate shots during the EMS procedure.

#### Detection of human EPCs in the temporalis muscle from EMS

Two weeks after EMS, the temporalis muscles were removed and subjected to IHC staining using Ku80 antibody (human-specific, Cell Signaling, #2180) to confirm the presence of the transplanted EPCs inside the muscular flap created via EMS.

### Microcirculation measurements

#### Craniotomy for observation of microcirculation

Two weeks after the EMS procedure, anesthetized rats were placed in a stereotaxic frame (Kopf Instruments, Tujunga, CA, USA). A 3 × 3 mm cranial window was opened medial to the EMS site and behind the coronal suture. The dura was opened carefully without injury to the brain surface under microscopic guidance.

#### Microvasculature density

Microcirculation was assessed by capillary videomicroscope: a CAM1 capillary anemometer (KK Technology, UK) with a high-resolution (752 × 582 pixels) monochrome charge-coupled device video camera was employed. A microscope was attached to a heavy support to allow three-dimensional adaptation without contacting the brain surface. The field of vision was 684 × 437 µm, and images were magnified to approximately 0.91 μm/pixel.

The microvasculature density was calculated as the number of vessels crossing a line divided by the total length of the line (De Backer’s score) [19]. A 20-μm cut-off point was used to separate small vessels from large vessels (mostly venules). Three to five 640 × 480-pixel images were taken per rat, and each image was divided by 6 × 4 lines. The numbers of vessels crossing the lines were counted and summed from the 3–5 images, and the average number of crossing points was then obtained to define the density of microcirculation. The data were expressed as n/mm^2^.

#### Regional blood flow, partial pressure of brain tissue oxygen (PbtO_2_), and temperature

The regional blood flow, PbtO_2_, and temperature were measured simultaneously in the same microregion of brain tissue using OxyFlo 2000E and OxyLite 2000E (Oxford Optronic Ltd, England). The region to be measured was located 1 mm medial to the temporalis muscle and just behind the coronal suture. The probe tip was inserted 2-mm-deep into the cortical surface.

### Functional measurement using the rotarod performance test

The rotarod test has been widely used to study motor learning after brain injury, including stroke and neurodegenerative diseases [20]. In this test, motor performances of rodents were analyzed by measuring latency to falling off a rod to evaluate their endurance, balance coordination, physical condition, and motor-planning [21]. The rotarod test was performed as described previously with some modifications [20]. Before BICAL surgery, the animals were trained for three consecutive days at a speed of 4 rpm, in three sessions per day for 5 min. Three weeks after BICAL surgery, rats were placed on the instrument (Panlab Rota Rod, Harvard Apparatus), under continuous acceleration (e.g., from 4 to 40 rpm for 600 secs), and the latency to fall was measured.

### Immunohistochemical (IHC) staining

Two weeks after various treatments, rats were killed by injection of pentobarbital (200 mg/Kg, i.p.) and perfused transcardially with 50 mL of saline followed by 500 mL of a fixative containing 4% paraformaldehyde in 0.1 M PBS at pH 7.3 for 30 min. IHC analysis was performed on cryostat sections or paraffin-embedded sections according to the requirement of each individual antibody: (1) serial coronal brain sections of a thickness of 6 µm were cut on a cryostat and mounted onto gelatin-coated slides for IHC staining, and (2) sections were de-paraffinized by heating at 60 °C for 30 min followed by xylene treatment, rehydrated by passing through a series of decreasing concentrations of ethanol (100%, 90%, 70%, and 50%) for 5 min per step and then, washed with 0.1 M PBS. Endogenous peroxidase was quenched with 3% hydrogen peroxidase for 10 min. The sections were incubated with 5% bovine serum albumin for 1 h to block non-specific background staining, then subsequently incubated with each primary antibody overnight at 4 °C and visualized using a Novolink Polymer Detection System (RE7140-K; Novocastra, Newcastle upon Tyne, UK). Antibodies against the following molecules were used in this study: pTau (Abcam, ab151559, 1:100), Ku80 (Cell signaling, #2180, 1:100), and TUNEL (Merck, QIA33).

### Western blotting

Proteins were isolated from the brain cortex of the experimental hemispheres and their concentrations determined using a BCA protein assay kit (Pierce). Twenty micrograms of protein were loaded onto 10% acrylamide SDS-gels. Following electrophoresis at 100 V for 1 h, proteins were transferred to PVDF membranes (Bio-Rad). Membranes were blocked in 5% fat-free dry milk dissolved in Tris-buffered saline (TBS), pH 8.0, plus 0.1% Tween-20 (TBS-T) for 1 h and probed overnight at 4 °C with the following antibodies at the designated dilutions: phospho-GSK3β (polyclonal antibody diluted 1:1000, Abcam), total-GSK3β (monoclonal antibody diluted 1:1000, Cell Signaling), phospho-Tau (polyclonal antibody diluted 1:500, Cell Signaling) and cleaved-caspase-3 (monoclonal antibody diluted 1:400, Sigma). GAPDH was used as a loading control. After rinsing with 0.5% TBS-T solution, the membranes were incubated with the secondary antibody, donkey anti-rabbit and anti-mouse antibody, conjugated with horseradish peroxidase (HRP) at a dilution of 1:20,000 for 1 h at room temperature. Signals were detected by enhanced chemiluminescence (ECL, Amersham) before exposure on radiographic film. The densities of stained bands were scanned and quantified using the Diversity One software package (PDI, NY, USA). To reduce differences between animals, at least three Western blots were performed at each time point for each animal. In addition, at least two or three repeated samples were always included in every set of experimental samples as internal standards.

### Statistical analysis

The distributional properties of the continuous variables were expressed as the mean ± SD. Normality was tested by the Shapiro–Wilk normality test. Comparisons of two groups were analyzed by Student’s *t*-test. One-way analysis of variance (ANOVA) followed by a Post Hoc Test (Tukey's multiple comparisons test) was performed for normally distributed data. The Kruskal–Wallis variance analysis test was employed for non-normally distributed data. A two-sided *p* value < 0.05 was considered statistically significant. Statistical analysis was performed using R 2.14.1 software (R Foundation for Statistical Computing, Vienna, Austria). Bar graphs were created using GraphPad Prism 7.0 software (GraphPad Software, USA).”

## Results

### EPC characterization

**Fig. 2:**
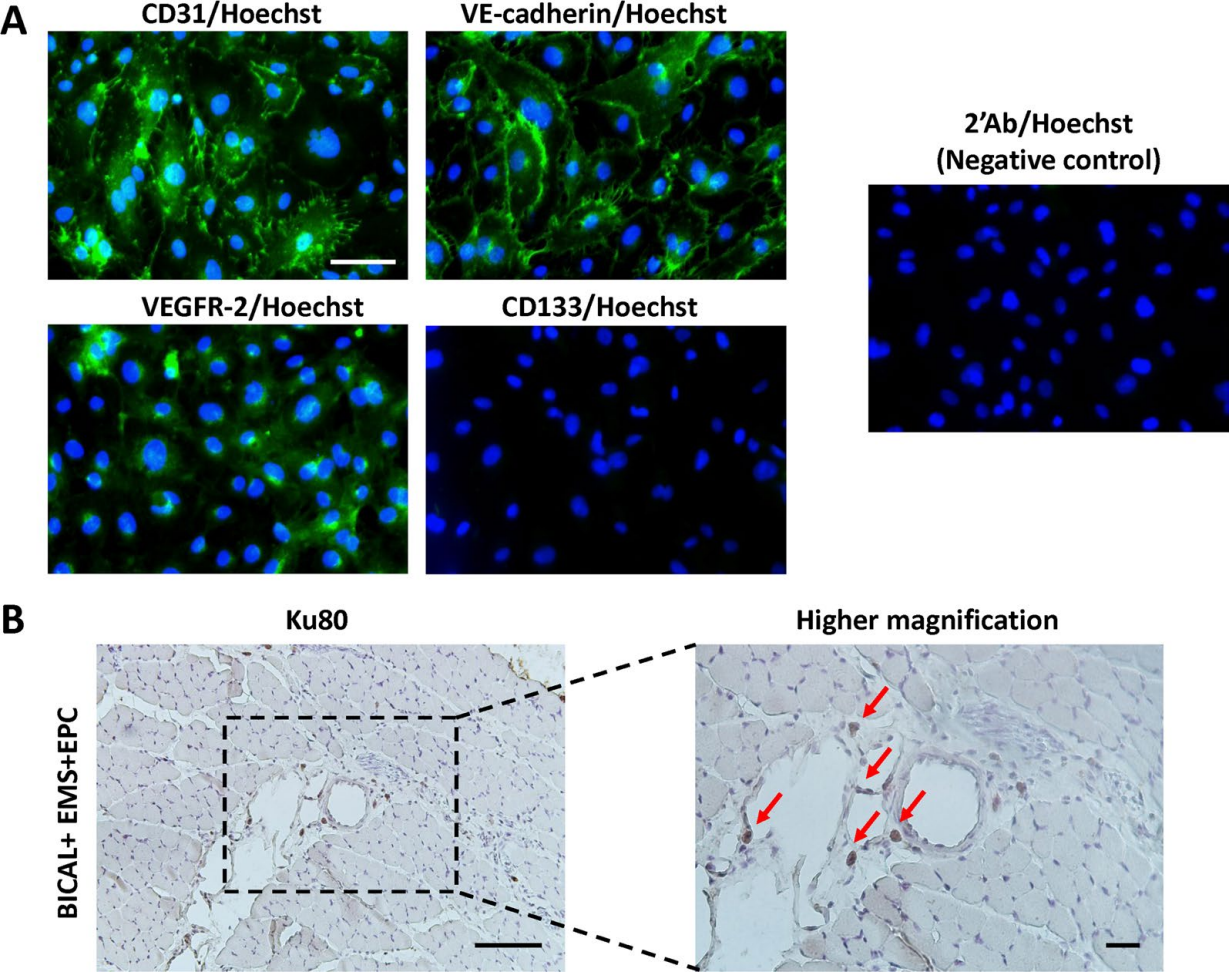
Characterization of human umbilical cord blood-derived late EPCs. **A** Late EPCs expressed endothelial markers CD31, VE-cadherin, and VEGFR-2, but did not express hematopoietic marker CD133 by immunofluorescence staining. The cell nuclei were counterstained with Hoechst 33,342. A negative control was incubated with a secondary antibody only. Cells were visualized using an inverted fluorescence microscope (HPF, 400×). Scale bars = 60 μm. **B**) Temporalis muscles were subjected to IHC staining with Ku80 antibody (human-specific). Representative IHC staining images show Ku80-positive cells (red arrows, HPF 400×) 2 weeks after transplantation, indicating survival of transplanted EPCs in vivo. Scale bars = 100 and 25 μm (left and right, respectively)

EPC markers CD31, VE-cadherin, and VEGFR-2 were strongly expressed in cultured cells, while hematopoietic marker CD133 did not display remarkable expression, suggesting highly purified EPC isolation (Fig. 2A). Temporalis muscles were subjected to IHC staining with Ku80 antibody (human-specific). Ku80-positive cells were detected from the temporalis muscle 2 weeks after EMS, indicating the survival of transplanted EPCs in vivo (Fig. 2B).

### The functional deficits of the BICAL group improved after EMS and EMS combined with EPC treatment

**Fig. 3:**
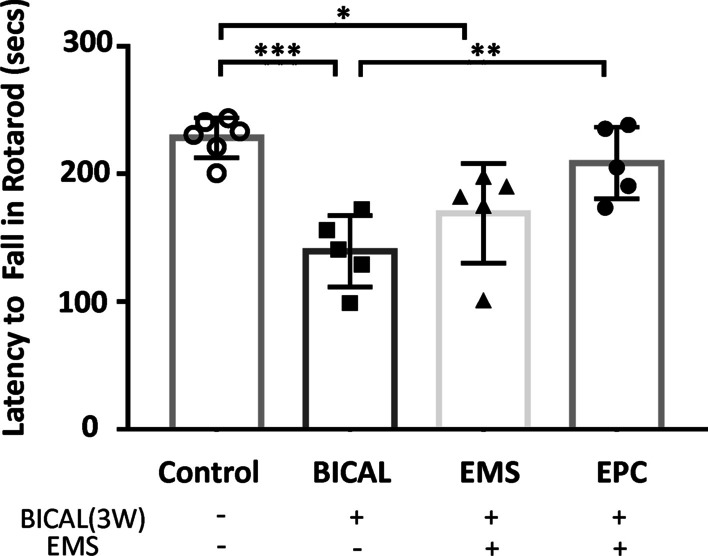
EMS combined with intramuscular injection of EPCs improved the functional outcome of BICAL rats. The latency to fall in the rotarod test was recorded, and a significant impairment in motor coordination performance was evident in the BICAL group as compared with the control group. The latency to fall was significantly improved in the EMS combined with EPC treatment group. *p < 0.05, **p < 0.01, ***p < 0.001, *n* = 5–6

The overall mortality rate after the surgery was 9.89% (9/91). As we observed that both EMS and EMS combined with EPC treatment resulted in amelioration of microcirculation impairment after BICAL, we further performed the rotarod test to assess functional outcome in these 2 groups after BICAL. The functional outcome of the BICAL and EMS group was significantly poorer than that of the control group (latency to fall duration in the rotarod test: BICAL vs. control, 139.38 ± 27.92 s vs. 228.28 ± 15.70 s, *p* < 0.001; EMS vs. control, 169.16 ± 39.05 s vs. 228.28 ± 15.70 s, *p* < 0.05). The EMS + EPC group showed a longer latency duration as compared with the BICAL group (EMS + EPC vs. BICAL, 208.63 ± 28.07 s vs. 139.38 ± 27.92 s, *p* < 0.01), while the EMS combined with EPC treatment group showed no difference compared with that in the control group (Fig. 3).

### Microcirculation impairment induced by BICAL and changes after EMS and various treatments

**Fig. 4:**
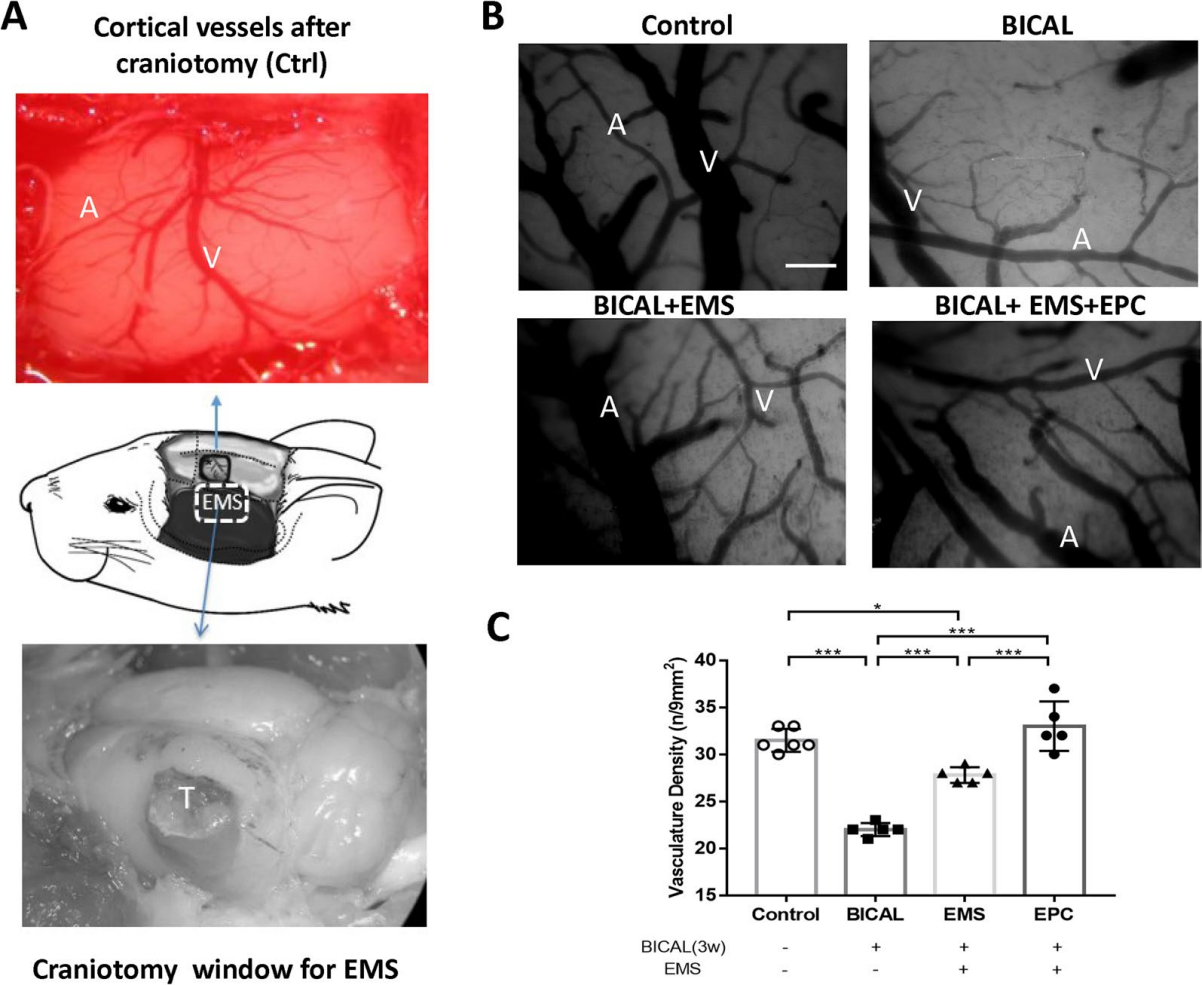
Effects of EMS and EMS combined with EPC treatment on microvasculature density after BICAL. **A** A 3 × 3 mm craniotomy window was opened for microvasculature and microcirculation measurement (upper panel, black line box). Two weeks after the EMS procedure (lower panel, white dotted box), the temporalis muscle, (marked as T) adhered to the brain surface well, indicating that the EMS procedure was successful. **B** Under videoscopic view, the vasculature, including the artery (a) and vein (v), was clearly seen on the brain surface. An obvious paucity of microcirculation on the cortical surface was noted 3 weeks after BICAL. **C** After quantification, microvasculature density decreased obviously after BICAL and was improved after EMS and EMS combined with EPC treatment. Scale bar = 100 μm. *p < 0.05, ***p < 0.001, *n* = 5–6

To investigate the effects of EMS and EMS combined with EPC treatment on vascular density, we performed a craniotomy behind the frontal suture at three weeks after BICAL (Fig. 4A). An obvious paucity of microcirculation on the cortical surface was noted by capillary videoscope in the BICAL rats, caused by constriction of the cortical arterioles. Under stagnation of blood flow, it was observed that the red blood cells (RBCs) in the venules were aligned in tandem fashion (Additional file 1: Video 1). Under videomicroscope, the vasculature density was markedly reduced 3 weeks after BICAL in the BICAL group, whereas the vasculature density was improved in both the EMS and EMS combined with EPC treatment group (Fig. 4B). After quantification, the vasculature density, expressed as n/mm^2^, was obviously decreased 3 weeks after BICAL (chronic BICAL vs. control, 23.5 ± 0.51 vs. 31.25 ± 0.77, *p* < 0.001; Fig. 4C). The microvasculature on the brain surface 2 weeks after EMS and EMS combined with EPC treatment demonstrated significant improvement in terms of vasculature density (EMS and EMS combined with EPCs vs. chronic BICAL, 28.58 ± 0.51 and 33.0 ± 1.18 vs. 23.5 ± 0.51, respectively, *p* < 0.001; Fig. 4C) and collateral circulation (Additional file 1: Video 1). Moreover, EMS combined with EPC treatment was more efficacious regarding the increased vasculature density than EMS after BICAL (*p* < 0.001; Fig. 4C).

**Fig. 5:**
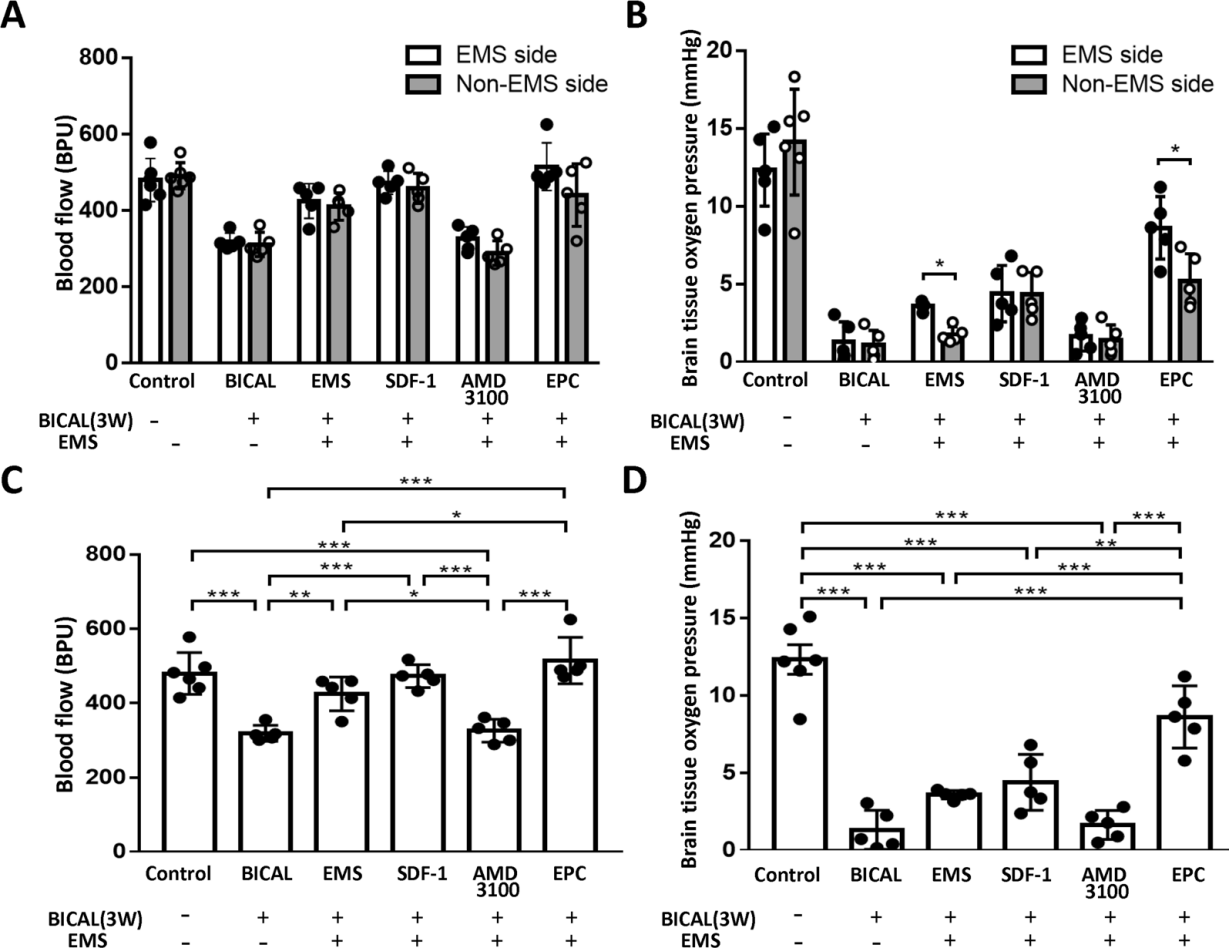
Effects of EMS and various treatments on cerebral microcirculation. **A** Regional blood flow and **B** partial pressure of brain tissue oxygen (PbtO_2_) of the control, BICAL, EMS and various treatment groups on the EMS (white bars) and non-EMS sides (gray bars) were measured simultaneously 3 weeks after BICAL. Comparison of the treatment efficacy between the EMS and non-EMS sides in each group was performed by the *t*-test. *p < 0.05. **C** On the EMS side, the reduction in regional blood flow induced by BICAL was rescued by EMS, EMS combined with SDF-1 or EPC treatment, but not by AMD3100 treatment. **D** EMS combined with EPC treatment significantly increased the BICAL-induced decrease in PbtO_2_. *p < 0.05, **p < 0.01, ***p < 0.001, *n* = 5–6

The regional blood flow, expressed in blood perfusion units (BPU), and partial pressure of brain tissue oxygen (PbtO_2_) of the various groups on the EMS and non-EMS sides were measured at 3 weeks after BICAL (Fig. 5A, B). There was no difference of regional blood flow between the EMS and non-EMS sides in various groups (Fig. 5A). Both EMS and the combination treatment of EPCs groups showed higher PbtO2 on the EMS than the non-EMS sides (*p* < 0.05; Fig. 5B). In the EMS hemispheres, regional blood flow was significantly decreased at three weeks after BICAL as compared with the control group on both the EMS (*p* < 0.001; Fig. 5C) and non-EMS sides (*p* < 0.001; Additional file 2: Fig. S1A) of the brain. In addition, EMS and the combination treatment of SDF-1 and EPCs resulted in a significant increase in regional blood flow as compared with the three weeks after BICAL group (*p* < 0.01–0.001; Fig. 5C), whereas only the EMS combined with EPC treatment group exhibited better blood flow than the EMS only group (*p* < 0.05; Fig. 5C). However, EMS combined with AMD3100 treatment moderately abrogated the SDF-1-mediated homing of EPCs effect and significantly decreased the cerebral blood flow as compared with the EMS combined with SDF-1 or EPC treatment groups (*p* < 0.001; Fig. 5C). Similar to the results obtained for regional blood flow, the PbtO_2_ was significantly decreased at three weeks after BICAL as compared with the control group on both the EMS (*p* < 0.001; Fig. 5D) and non-EMS sides (*p* < 0.001; Additional file 2: Fig. S1B). However, among the various treatment groups, only EMS combined with EPC treatment resulted in the greatest augmentation of PbtO_2_ on the EMS side as compared with the three weeks after BICAL group (*p* < 0.001; Fig. 5D). Moreover, on the EMS side, EMS combined with EPCs was more efficacious in increasing PbtO_2_ than any other treatment after BICAL (*p* < 0.01–0.001; Fig. 5D).

### Phosphorylation of Tau protein induced by BICAL and changes after EMS and application of SDF-1, AMD3100, and EPCs

**Fig. 6:**
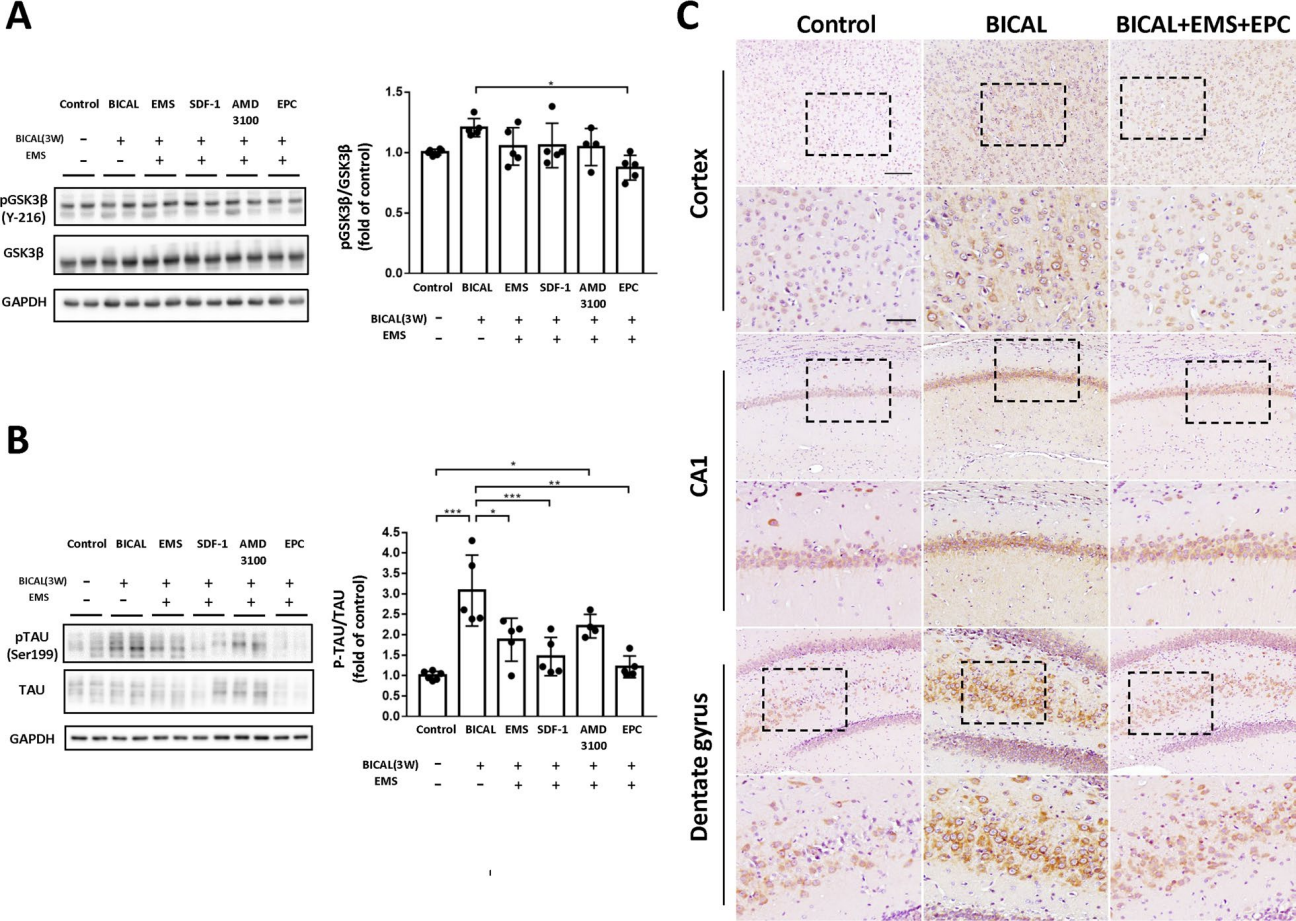
EMS combined with EPC treatment protects the rat brain against BICAL-induced Tau phosphorylation. Representative immunoblot (left) and quantification (right) of **A** pGSK3β (Y216) and **B** pTau (Ser199) in the control, BICAL, and treatment group rats. EMS combined with EPC treatment significantly decreased the pGSK3β/GSK3β and pTau/Tau ratios as compared with the BICAL group. **C** Representative microphotography showing immunoexpression of pTau (Thr231) (shown in brown) in the cerebral cortex, hippocampus CA1, and dentate gyrus (DG) regions of the control, BICAL, and EMS combined with EPC treatment rats. *p < 0.05, **p < 0.01, ***p < 0.001, *n* = 4–6. Scale bars = 100 µm (upper panel) and 50 µm (lower panel)

As it has been shown that Tau phosphorylation is regulated by GSK3β activity [22], we determined the effects of EMS combined with EPC treatment on phosphorylation of GSK3β (Y216) and Tau (ser199) by Western blot analysis 3 weeks after BICAL induction. The WB data showed that only EMS combined with EPC treatment significantly decreased the pGSK3β (Y216)/GSK3β ratio as compared with the BICAL group (Fig. 6A, *p* < 0.05). In addition, pTau (Ser199) was significantly increased in the BICAL and AMD3100 groups relative to the controls (Fig. 6B). The EMS, SDF1 and EPC groups showed a significantly decreased pTau (Ser199)/TAU ratio as compared with the BICAL group (Fig. 6B). We further examined the distribution of Tau phosphorylation (Thr231) in the rat brain by IHC staining. The IHC results showed a robust, diffuse pattern of pTAU (Thr231) in the cortex, hippocampus CA1 and DG regions of the BICAL rats as compared with the controls (Fig. 6C). However, EMS combined with EPC transplantation markedly decreased the level of pTau (Thr231) as compared with the BICAL rats. These findings suggested that EMS combined with EPC transplantation reduced phosphorylation of GSK3β (Y216), contributing to a decrease in the activity of this kinase, ameliorating the pTau level.

### EMS combined with EPC treatment reduced BICAL-induced apoptosis in the cortex

**Fig. 7:**
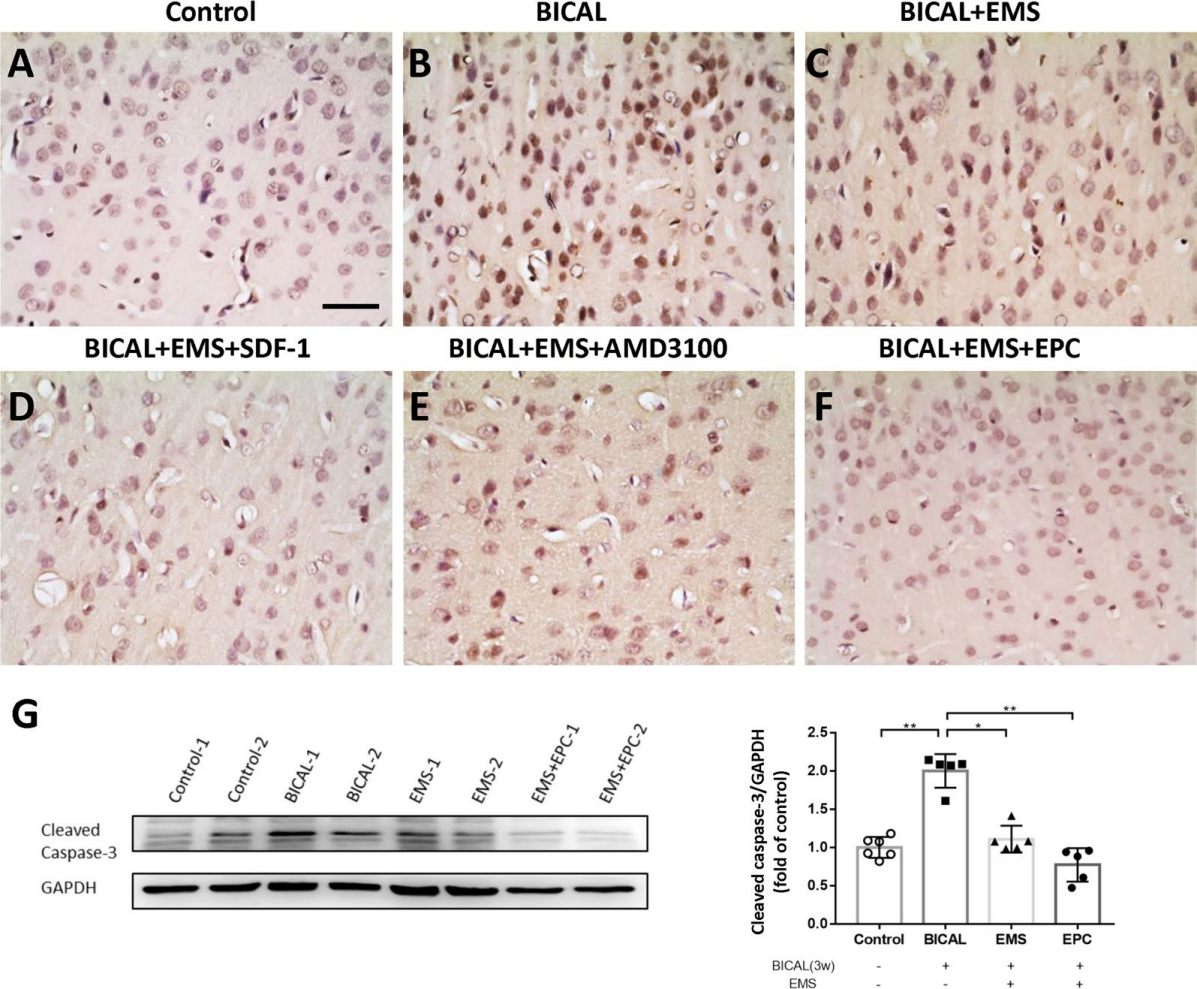
EMS combined with EPC treatment decreased the apoptosis induced by BICAL. Coronal brain sections were subjected to TUNEL staining (control, **A**). EMS (**C**), EMS combined with SDF-1 (**D**), and EMS combined with EPC (**F**) treatments remarkably mitigated the severe cell death induced by BICAL (**B**), whereas EMS combined with AMD3100 treatment did not (**E**). (**G**)Western blot results showed that cleaved-caspase-3 was significantly increased after BICAL. EMS and EMS combined with EPC treatment reduced the level of cleaved-caspase-3. GAPDH staining was used as the protein loading control. *p < 0.05, **p < 0.01, *n* = 5–6. Scale bars = 50 µm

To examine the apoptotic cells in cortical neuronal cells, we performed TUNEL staining and WB analysis for cleaved-caspase-3 protein. As compared with the control group, TUNEL-positive cells were increased in the BICAL and AMD3100 groups (Fig. 7A, B and E), while TUNEL-positive cells were markedly decreased in the EMS, EMS plus SDF-1, and EMS plus EPC groups after BICAL (Fig. 7C, D and F). A quantitative study using WB demonstrated elevation of the cleaved-caspase-3 level after BICAL as compared with the control group (*p* < 0.01; Fig. 7G). The level of cleaved-caspase-3 decreased after EMS and was further decreased in the EPC group (*p* < 0.05 and *p* < 0.01 vs. 3 weeks BICAL, respectively; Fig. 7G).

## Discussion

Angiogenesis is defined as the growth of new vessels from pre-existing ones, whereas arteriogenesis comprises remodeling of pre-existing arterio-arteriolar anastomoses into completely developed and functional arteries [23–25]. Collateral circulation vessel induction by indirect revascularization is considered a kind of angiogenesis. Previous studies of EPCs in MMD have presented contradictory data, which included increased amounts of EPCs [26–28], decreased amounts of EPCs [29], and defective EPC function [27, 29]. Reduced numbers and impaired function of circulating EPCs have been shown to be related to endothelial dysfunction, cerebral infarction, and coronary artery disease [5, 7, 30, 31]. Meanwhile, studies have identified a subset of outgrowth cell populations with an endothelial phenotype and vasculogenic potential after long-term culture of peripheral blood mononuclear cells (PBMNCs) [32–34]. EPCs thus have been implicated in neoangiogenesis and are capable of proliferating and differentiating into endothelial cells for vascular regeneration [8], and play a role in the maintenance of cerebral blood flow.

Several factors have been shown to activate EPCs, such as hypoxia-inducible factor-1α (HIF), VEGF, erythropoietin, SDF-1, and nitric oxide [14, 35]. On the other hand, EPCs secrete various proangiogenic growth factors, which may be beneficial in chronic cerebral ischemia [36, 37]. These proangiogenic factors include VEGF, hepatocyte growth factor (HGF), transforming growth factor-β, and SDF-1, as well as neurotrophic and neuroregulatory cytokine brain-derived neurotrophic factor (BDNF) [38]. In summary, EPCs may play a role in maintaining vascular homeostasis and cerebral blood flow and could be beneficial for angiogenesis and vascular regeneration.

There is growing evidence to demonstrate the substantial clinical and pathophysiological similarities between chronic cerebrovascular disease and MMD. A series of models of chronic hypoperfusion, including bilateral CCA or ICA occlusion or microcoil methods of chronic ischemic models, has been developed to evaluate the microcirculation changes in response to ischemia and the benefits of surgical revascularization [16, 39, 40]. Although there exist discrepancies in the histology and pathogenesis of the vascular pathology between these hypoperfusion models and MMD pathophysiology, they play a vital role in the understanding and treatment of MMD. The effect of EMS, which has been the most commonly used indirect revascularization procedure for MMD patients, was also evaluated in these hypoperfusion models [39, 40]. Therefore, we applied EMS combined with EPC treatment as a potent new combination therapy for MMD. To improve accessibility to ischemic cerebrovascular disease, the other common recanalization procedure for carotid artery disease combined with EPC treatment should be considered in future study.

In the present study, we successfully established an animal model of chronic cerebral ischemia, which was induced by ligation of bilateral ICAs. The cerebral ischemic status was confirmed by various microcirculation measurements, including vascular density, regional blood flow, and PbtO_2_. We also demonstrated that EMS, a commonly used indirect revascularization process in clinical practice, with the application of EPCs or SDF-1 improved the cerebral hypoperfusion by reversing the impairments of vascular density, regional blood flow, and PbtO_2_ in rats. Moreover, only EMS combined with EPC treatment resulted in significantly increased regional blood flow and PbtO_2_ as compared with EMS treatment only, indicating a possible therapeutic efficacy in the prevention of BICAL-induced microcirculation impairment. Although the mechanism by which the EPCs affect the indirect revascularization remains unclear, we speculated that during the EMS procedure, the dura and arachnoid membranes are opened so that the ischemic signals transmit directly through contact with the vascularized donor flap to the systemic circulation. The signal activates and homes the EPCs from bone marrow to the ischemic site, and the factors secreted by EPCs further induce angiogenesis at the synangiosis site. This speculation was supported by evidence that direct intramuscular injection of EPCs further improved the regional cerebral blood flow and PbtO_2_ as compared with the effect of EMS only. The addition of SDF-1 into the TISSEEL, which was pasted between the cortical vessels and the muscular flap, further improved the microcirculation of the cortical vessels, whereas the addition of AMD3100, an antagonist of SDF-1, worsened the angiogenesis effect of EMS. These findings suggested a pivotal role of EPCs in indirect revascularization.

It was interesting to note that the microcirculation of the contralateral non-revascularized side was also improved significantly after EMS. The application of SDF-1 and EPCs further enhanced the therapeutic effects of EMS, respectively. These findings implied that indirect revascularization not only provides a bridge for direct angiogenesis formation, but also exerts an angiogenesis effect on the contralateral side through a distinct paracrine mechanism. EPCs may participate in the secretion of those paracrine factors.

The vascular endothelial dysfunction following chronic cerebral ischemia may cause brain damage and neurodegeneration via several mechanisms, which include induction of amyloid accumulation, Tau hyperphosphorylation, neuronal loss, and neuro-inflammation [41, 42]. It has been reported that EPC transplantation mitigated the increases in the levels of hippocampal Tau phosphorylation and its upstream GSK-3β in an experimental model of Alzheimer’s disease [43]. In this study, we demonstrated that the levels of phosphorylated Tau protein and caspase-3 were significantly increased after BICAL, while EMS ameliorated the accumulation of Tau protein and apoptosis in the ischemic brain. Direct injection of EPCs into the temporalis muscle further decreased the amounts of phosphorylated Tau and GSK3β (Y216) protein and the level of caspase-3, in line with previous research [43]. GSK-3β is the main isoform of GSK3 and is highly expressed in neurons [44]. Studies have shown that increased pGSK3βY216 enders the enzyme activity and contributes to phosphorylation at several sites of Tau (Ser396/Ser199/Thr231/Ser404/Thr205) [45–47]. However, the mechanism underlying the effect of EMS combined with EPC treatment in terms of decreasing pGSK3β (Y216) caused by BICAL requires further investigation. In addition, EMS plus SDF-1 obviously decreased the amounts of phosphorylated Tau and caspase-3, whereas AMD3100 exacerbated Tau phosphorylation and increased the number of TUNEL-positive cells. The above findings suggested that EPCs not only contribute to angiogenesis after indirect revascularization, but also exert a neuroprotective effect. On the other hand, previous studies suggested several non-apoptotic functions of cleaved-caspase-3 expression after stroke, which were predominantly associated with the post-stroke inflammatory response, such as reactive astrogliosis and immune cell infiltration (macrophage/microglia) [48]. Although this may reflect an overestimation of apoptosis, it also indicates an inflammatory reaction in glial cells in response to cerebral ischemia.

Our study results showed that the effects of EMS and various treatments on the non-EMS side were similar to those on the EMS side. We propose that EPCs and SDF-1 may mediate the improvement of the microcirculation of the non-EMS side via the cerebrospinal fluid, though we have no direct evidence of this. However, there were some discrepancies in the improvement of regional blood flow and PbtO_2_ caused by the addition of SDF-1; these may be due to the number and biological variation of animals or some mechanisms still not known. In addition, the early arterial recanalization has been considered as a good prognosis for functional outcome after ischemic stroke, especially in acute stroke [49]. According to the preclinical recommendations of the Stroke Therapy Academic Industry Roundtable (STAIR), highly effective reperfusion with thrombectomy could be employed in conjunction with neuroprotective agents to facilitate the successful translation stroke therapies [50, 51]. Indirect revascularization combined with EPC treatment might concurrently provide the optimal therapeutic potential. Further studies are mandatory to elucidate the detailed mechanism of neuroprotection.

Additionally, our animal model of chronic cerebral hypoperfusion generated by BICAL surgery reflected impaired cerebral small vessels and microcirculation impairment, both of which are associated with a poor outcome in stroke patients [52, 53]. Cerebral small vessel disease has been reported as a leading cause of cognitive dysfunction, vascular dementia and functional impairment in elderly patients [54]. By using the rotarod task, we demonstrated the BICAL-induced microcirculation impairment might not only cause motor dysfunction but also contribute to cognitive impairment in motor learning. On the other hand, growing evidence has also shown that interactions between risk factors, including aging, hypertension, diabetes, genetic, cellular/molecular factors, etc., boost the development and progression of cerebral small vessel disease and cognitive deficits [55–57]. Thus, future studies investigating comorbid animal models and cognitive function in a longer-term manner are needed to clarify this issue and may possibly accelerate the development of novel therapeutic strategies.

## Conclusion

An animal model of chronic cerebral ischemia was successfully established, followed by indirect revascularization via EMS to the ischemic brain. We demonstrated that intramuscular injection of EPCs played a key role in augmenting the effect of indirect revascularization. The addition of chemotaxis factor SDF-1, and its antidote AMD3100, further supported the pivotal role of EPCs in the process of synangiosis. The combination of EPC application and surgical treatment may shed some light on the optimal treatment of chronic cerebral ischemia.

## Supplementary Information

**Additional file 1**: **Video 1**. An obvious paucity of microcirculation on the cortical surface was noted by capillary videoscopy 3 weeks after bilateral internal carotid artery ligation (BICAL). Stagnation of venous Red blood cells (RBCs) and collateral circulation disappeared intermittently. The vasculature density of the encephalo-myo-synangiosis (EMS) side improved after BICAL. The vasculature density of the non-EMS side also showed significant improvement after BICAL.

**Additional file 2**: **Fig S1**. Effects of EMS and various treatments on cerebral microcirculation of the non-EMS side. **A** Regional blood flow and **B** partial pressure of brain tissue oxygen (PbtO_2_) were measured simultaneously 2 weeks after EMS on the non-EMS side. The non-EMS side had similar changes to EMS side after various treatments. Bars on graphs are mean ± SD. *p < 0.05, *n *= 5–6.

## Data Availability

The data and materials that support the findings of this study are available from the corresponding authors upon reasonable request.

## Abbreviations

EPC: Endothelial progenitor cell
BICAL: Bilateral internal carotid artery ligation
EMS: Encephalo-myo-synangiosis
EDAS: Encephalo-duro-arterio-synangiosis
MMD: Moyamoya disease
SDF-1: Stromal cell-derived factor 1
PbtO_2_: Partial pressure of brain tissue oxygen
pTau: Phosphorylated tau
pGSK-3β: Phosphorylated glycogen synthase kinase 3 beta
TUNEL: Terminal deoxynucleotidyl transferase dUTP nick end labeling
CCA: Common carotid artery
ICA: Internal carotid artery
MNC: Mononuclear cell
EBM-2: Endothelial cell basal medium-2
EGM-2: Endothelial cell growth medium-2
PBS: Phosphate-buffered saline
VEGF: Vascular endothelial growth factor
VE-cadherin: Vascular endothelial-cadherin
CD31: Cluster of differentiation 31
IHC: Immunohistochemical
i.p.: Intraperitoneally
TBS: Tris-buffered saline
GAPDH: Glyceraldehyde-3-phosphate dehydrogenase
BPU: Blood perfusion unit
BDNF: Brain-derived neurotrophic factor
PBMNCs: Peripheral blood mononuclear cells
HGF: Hepatocyte growth factor
