# Supplementary figures and images for: Combination of indirect revascularization and endothelial progenitor cell transplantation improved cerebral perfusion and ameliorated tauopathy in a rat model of bilateral ICA ligation
Source: Stem Cell Res Ther. 2022 Nov 12;13:516. doi: 10.1186/s13287-022-03196-1 (PMC9652785; doi:10.1186/s13287-022-03196-1)

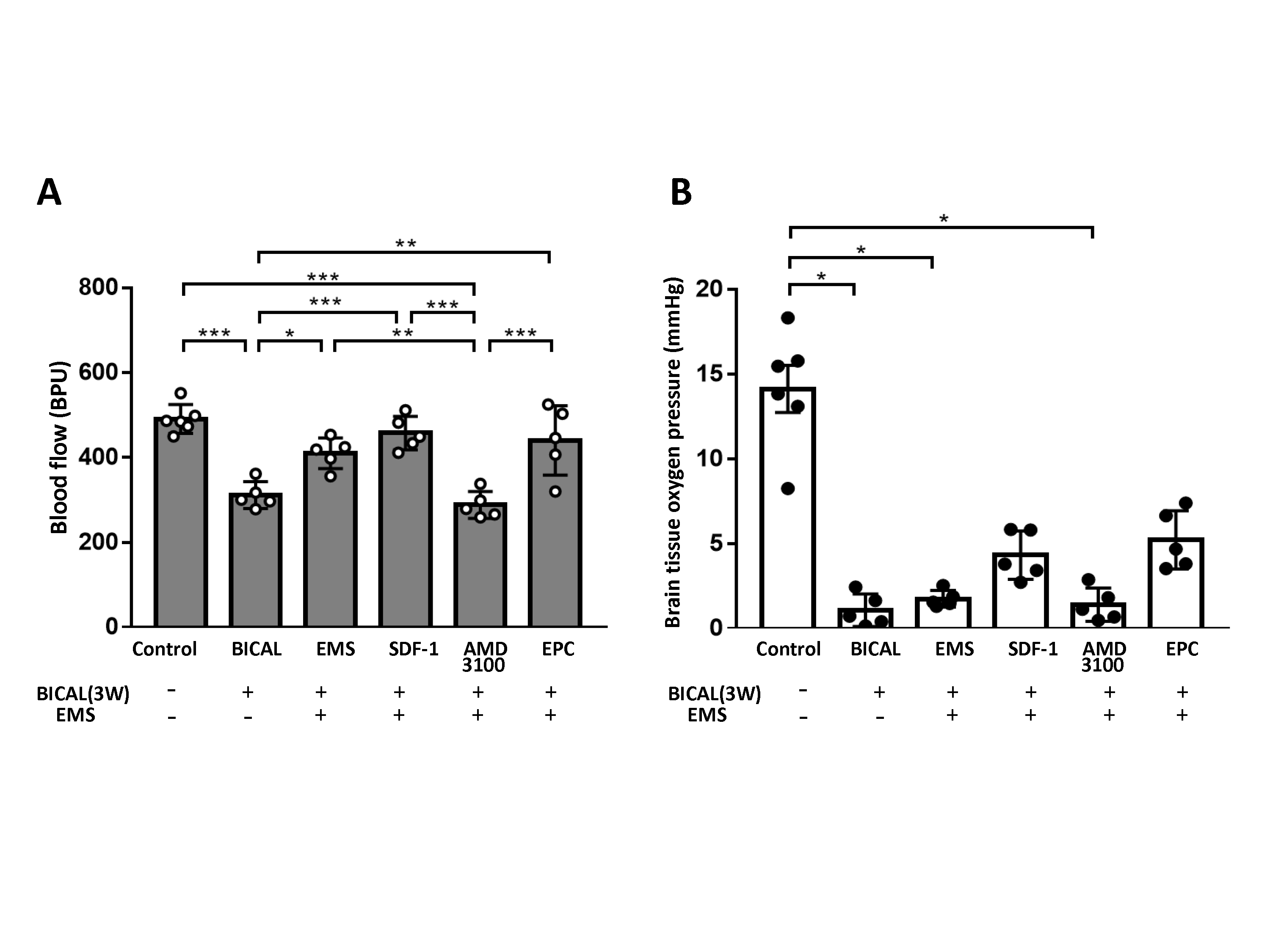

Supplement: Supplementary file 2 — Additional file 2: Fig S1. Effects of EMS and various treatments on cerebral microcirculation of the non-EMS side. A Regional blood flow and B partial pressure of brain tissue oxygen (PbtO2) were measured simultaneously 2 weeks after EMS on the non-EMS side. The non-EMS side had similar changes to EMS side after various treatments. Bars on graphs are mean ± SD. *p < 0.05, n = 5–6. [file 13287_2022_3196_MOESM2_ESM.tif]
